# Surface Modification with Particles Coated or Made of Polymer Multilayers

**DOI:** 10.3390/pharmaceutics14112483

**Published:** 2022-11-16

**Authors:** Konstantinos T. Kotoulas, Jack Campbell, Andre G. Skirtach, Dmitry Volodkin, Anna Vikulina

**Affiliations:** 1 School of Science and Technology, Nottingham Trent University, Clifton Lane, Nottingham NG11 8NS, UK; 2 Bavarian Polymer Institute, Friedrich-Alexander-Universität Erlangen-Nürnberg, Dr.-Mack-Straße 77, 90762 Fürth, Germany; 3 Bio-Nanotechnology Laboratory, Faculty of Bioscience Engineering, Ghent University, 9000 Ghent, Belgium

**Keywords:** layer-by-layer, polyelectrolyte multilayers, immobilization, coatings

## Abstract

The coating of particles or decomposable cores with polyelectrolytes via Layer-by-Layer (LbL) assembly creates free-standing LbL-coated functional particles. Due to the numerous functions that their polymers can bestow, the particles are preferentially selected for a plethora of applications, including, but not limited to coatings, cargo-carriers, drug delivery vehicles and fabric enhancements. The number of publications discussing the fabrication and usage of LbL-assembled particles has consistently increased over the last vicennial. However, past literature fails to either mention or expand upon how these LbL-assembled particles immobilize on to a solid surface. This review evaluates examples of LbL-assembled particles that have been immobilized on to solid surfaces. To aid in the formulation of a mechanism for immobilization, this review examines which forces and factors influence immobilization, and how the latter can be confirmed. The predominant forces in the immobilization of the particles studied here are the Coulombic, capillary, and adhesive forces; hydrogen bonding as well as van der Waal’s and hydrophobic interactions are also considered. These are heavily dependent on the factors that influenced immobilization, such as the particle morphology and surface charge. The shape of the LbL particle is related to the particle core, whereas the charge was dependant on the outermost polyelectrolyte in the multilayer coating. The polyelectrolytes also determine the type of bonding that a particle can form with a solid surface. These can be via either physical (non-covalent) or chemical (covalent) bonds; the latter enforcing a stronger immobilization. This review proposes a fundamental theory for immobilization pathways and can be used to support future research in the field of surface patterning and for the general modification of solid surfaces with polymer-based nano- and micro-sized polymer structures.

## 1. Introduction

**Figure 1:**
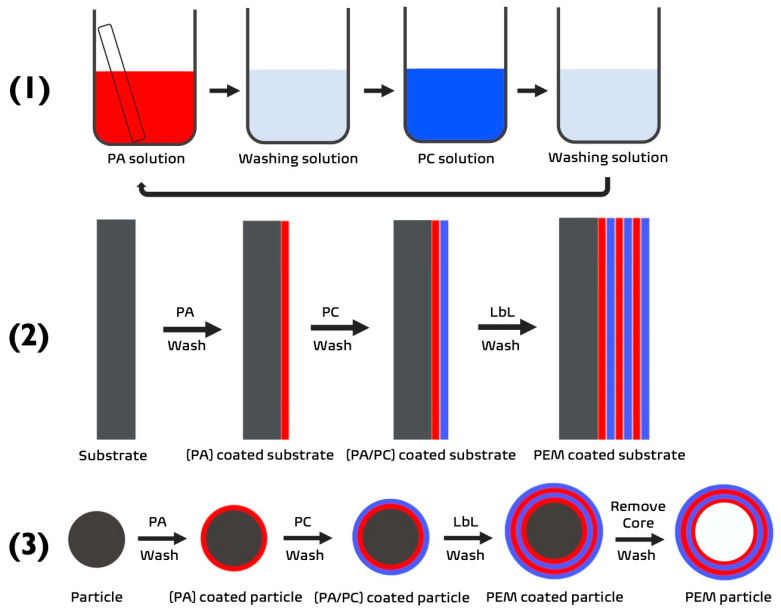
(**1**) Schematic demonstrating the process of LbL via dip-coating. (**2**,**3**) The build-up of a PEM film upon a substrate to form a (**2**) 2D PEM or (**3**) 3D PEM-coated or PEM particle. PA—polyanion (red), PC—polycation (blue).

Over the previous three decades, LbL assembly technologies have evolved and adapted in accordance with the materials used, and the products desired. Conventional polymer LbL assembly is based on the adsorption of oppositely charged polymers on to a surface (Figure 1) via forces where entropic and enthalpic interactions play a governing role [1,2]. Many modifications have been implemented to conventional LbL assembly during the intervening years to enhance the properties of the sequentially deposited layers. This has resulted in new branches of LbL assembly, such as 3D bio-printing (unconventional LbL) and saloplastics (quasi-LbL) [1]. As a result of this, LbL assembly is now prevalent in many fields because of its ability to coat an array of different surfaces, regardless of the topography and geometry.

Significant research has been conducted on polyelectrolyte multilayers (PEMs) deposited on to particles via LbL. The polyelectrolytes used can be biodegradable, biocompatible, and can possess various (bio)properties, including anti-inflammatory and osteogenic properties [3]. Consequently, these particles have a plethora of applications, including but not limited to delivery devices, biomedical coatings, protective coatings, and photocatalysis [4,5,6,7,8,9,10].

When fabricating these particles via electrostatic LbL deposition, the predominant interactions that influence the interfacial assemblies are ion pairing, and other interactions such as: van der Waals forces, guest-host interactions, base-pair interactions, hydrogen bonding, hydrophobic interactions, and polar interactions [11,12,13,14,15]. What separates multi-layered particles in their corresponding fields, is the fact that compared to other systems, the decomposable cores used to template multilayer structures can be synthesised from numerous components such as organic particles, biomolecules, polymers and inorganic salts [9,16,17]. To illustrate, mesoporous vaterite CaCO_3_ particles (commonly used as delivery devices) are frequently used for PEM particle preparation [18,19], and may be prepared from the inorganic salts CaCl_2_ and Na_2_CO_3_ [20], ranging from submicron to tens of microns in size [21]. Moreover, for these particles, the vaterite core is mesoporous [3], allowing for the loading of drugs and other bioactive molecules into its pores prior to the formation of the PEMs on the particle’s surface [4,6,17].

Another key aspect is the inherent structure of these particles. Since the LbL assembly takes place on the surface of the template (core), its morphology, and hence its properties, can be further modified by dissolution of its original core, impacting the cargo release properties [22,23]. Such properties may be further modified by the deposition of metal nanoparticles into the PEMs for catalysis or anti-corrosion coatings, for example [6,7,24,25,26]. The ability to functionalise such PEM particles provides a huge scope for potential applications, including recent biomedical applications (e.g., radioprotectants and in vivo imaging) [27,28].

When dissolving the core, the cargo it has encapsulated is now solely surrounded by the PEM, forming a shell (capsule structure). If the core is not dissolved, the structure is a PEM-coated particle. This provides the particle with a stimuli-responsive barrier, permitting cargo-releasing techniques based on the decomposition of the PEMs at a desired site and time [4,6,9,17]. The inclusion of inorganic cores minimizes the osmotic pressure and LbL shell decomposition during their dissolution [22], as only ions remain after the core dissolution, which can then freely diffuse outside. The two main release categories are: (a) chemical, via a change in pH or a change in ionic strength [29,30], or, (b) physical, via thermal triggers (electrical field [31], IR-light [7], laser [32], alternating magnetic field [15]).

This review addresses how LbL-assembled PEM particles are immobilized onto solid surfaces by providing answers as to which factors influence the immobilization of the particles, alongside which techniques are used to confirm their adhesion on to solid surfaces. The deposition of PEM particles has been cited amply in the past, however, reports mostly focus on the particle’s properties, biocompatibility, and subsequent applications, neglecting to address in detail how they adhere to a solid surface. This review, therefore, aims to combine the current literature to establish a general theory that can be used to assist future research on the immobilization of PEM particles on to solid surfaces.

## 2. Factors Influencing PEM Particle Immobilization

### 2.1. Forces

One of the most straightforward methods for immobilizing particles on solid surfaces is accomplished by surface-patterning [33]. Surfaces possessing the same charge repel each other, whereas, oppositely charged surfaces are attracted electrostatically [33]. When considering these relationships between two surfaces, in combination with the distance between them, we are introduced to the Coulombic force (electric force). Electrostatic attraction plays a significant role in the immobilization of particles on to a patterned surface and can complement their covalent binding to it [33].

Specifically, adhesion that is dominated by covalent bonding is referred to as chemical immobilization, whereas non-covalent-based mechanisms are referred to as physical. The charge modification of a surface also influences the capillary force. This force arises from adhesion and cohesion-induced pressure, between a particle’s and a liquid’s surface. The capillary force also plays a key role in guiding PEM particles into their final immobilized position [33]. Nonetheless, it is the particle’s shape that determines the degree of influence that the capillary force has on its immobilization. In detail, the shape of a PEM particle is dependent on the core and polymers used. For instance, MnCO_3_ PEM particles are cubic whereas vaterite CaCO_3_ PEM particles are spherical; the differentiating factor between the two is the core since their PEMs are formed from the same polymers, polyallylamine hydrochloride (PAH) and polysodium 4-styrenesulfonate (PSS) [34].

**Figure 2:**
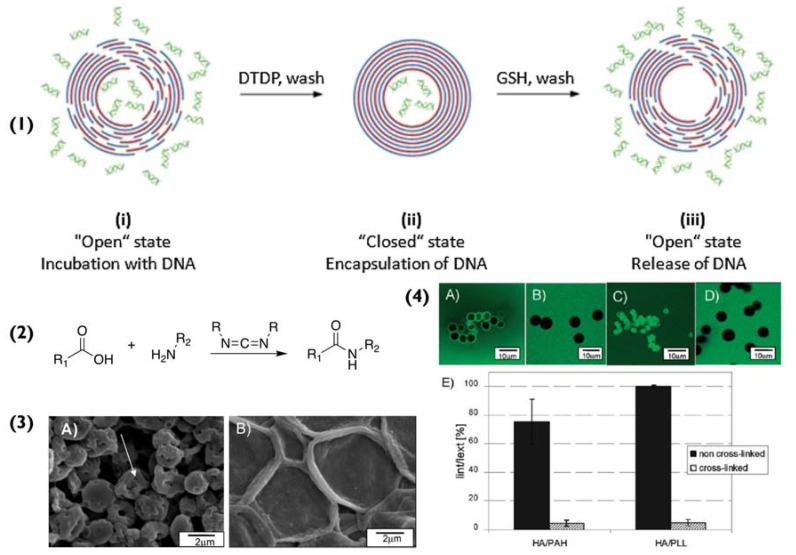
(**1**) Schematic for the encapsulation and release of DNA within multilayer capsules. (**i**) capsules are incubated with DNA, (**ii**) followed by treatment with 2,2′-dithiodipyridine (DTDP) to form disulphide bonds and to “close the capsules”, effectively trapping the DNA. (**iii**) The capsules are treated with glutathione to reduce the disulphide groups and release the DNA. Reprinted with permission from reference [39] copyright © 2014 Elsevier. (**2**) Chemical equation for the chemical cross-linking of carboxylic acids and amine groups with EDC, (**3**) (**A**) pristine (HA/PLL)_4.5_ capsules and (**B**) the effect of cross-linking with 200 mM EDC. (**4**) The effect of cross-linking on the permeability of (HA/PAH)_4.5_ and (HA/PLL)_4.5_ capsules. Confocal images of pristine (**A**,**C**) and crosslinked (**B**,**D**) (HA/PAH)_4.5_ and (HA/PLL)_4.5_ capsules incubated in 4 kDa dextran labelled with fluorescein isothiocyanate (FITC) solution. (**E**) comparison of the permeability of (HA/PAH)_4.5_ and (HA/PLL)_4.5_ capsules in 0.02 M MES buffer (pH 6.5). Permeability is expressed as the ratio of fluorescence intensities of the capsules interior (Iint) and surrounding solution (Iext) 20 min after mixing capsules and solutions of 4 kDa dextran-FITC. Adapted with permission from reference [38] copyright © 2010 American Chemical Society.

Equally important however are the polymers that coat the particles and the solid surfaces, since they determine how, and to what extent, the van der Waal forces will influence the immobilization [35]. The most dominant dipole–dipole interactions in van der Waal forces are hydrogen bonds. Van der Waal forces are weak, however, in the case of a coated surface (platform or particle), the larger the surface contact area, the greater the van der Waal interactions. When a particle is coated with PEMs, its subsequent bilayers are held in place via electrostatic attraction and hydrogen bonds, formed between each polymer’s backbone. Both interactions help stabilize the system and are the main reason as to why the PEM particles do not degrade during core dissolution. Having said that, in some cases, when the particles are in extreme pH or high ionic strength environments, these two interactions are not sufficient to preserve the particle’s stability [36]. In these scenarios, cross-linking is used to enhance the stability of PEM particles and improve their mechanical strength [37], as wells determine their permeability [38], as illustrated in Figure 2(4). Common examples of cross-linking include disulphide bonding [39] (Figure 2(1)), 1-ethyl-3-(3-dimethylaminopropyl) carbodiimide (EDC) [38], and glutaraldehyde [40]. Cross-linking is prevalent in biomedical applications, to ensure that the PEM-particles do not disassemble at physiological conditions [41,42].

Hydrogen bonds can also form between the outermost polymer layer on the particle and the solid surface, consequently aiding immobilization. An example of this would be hydrogen bonding between polycationic PAH and polyanionic PSS via the corresponding amino and oxygen ends [43]. The hydrogen bonds formed are highly dependent on pH, temperature, and salt concentration. For example, ionizing carboxylic groups can disrupt the hydrogen bonds they form and consequently the system’s stability [44]. For instance, poly(N,N-dimethylaminoethyl-methacrylate) (PDMAEMA) hydrogen bond cross-linking to sodium alginate (SA) is disrupted when ionized, since there is a lower amount of tertiary amino groups available [45]. Varying ionic strength has also been linked to particle shrinkage; an example being the PSS/poly diallydimethylammonium chloride (PDADMAC) [46].

**Figure 3:**
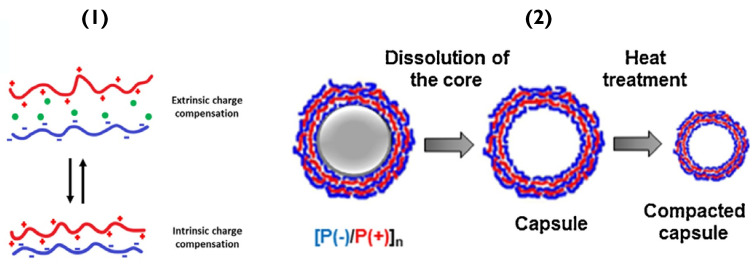
(**1**) Schematic of (bottom) PEMs containing only polymer-polymer interactions (intrinsic compensation) and (top) overcompensated films with polymer-counterion interactions (extrinsic compensation). Reprinted with permission from reference [3] copyright © 2021 American Chemical Society. (**2**) Schematic of PEM capsule formation and their thermal treatment. Reprinted with permission from reference [50] copyright © 2018 Elsevier.

Altering the salt concentration used when forming the PEMs can also lead to similar disruption, resulting in poorly defined layers with many defects; the disruption being most impactful for the electrostatic interactions. Specifically, in low salt concentrations, the PEM systems are controlled enthalpically since salt adds more entropy to a system. Increasing salt concentration can affect polyelectrolyte chain mobility and shell permeability [47]. At local chain dynamics, there are two scenarios: (a) extrinsic charge compensation, where the number of counter ions from salt (e.g., Na^+^ and Cl^−^) is significant and charge compensation is due to mainly counterions interacting with permanent charges of polymers or, (b) intrinsic charge compensation, which incorporates mostly ion pairs and little free charges (Figure 3(1)) [48]. This equilibrium allows the PEM to arrange preferentially. The best thermodynamic condition is, of course, intrinsic compensation, as every charge on the polymer backbone is connected to its counterpart.

In a water-based system, increasing temperature decreases the degree of hydrogen-bonding [44]. In other cases, such as hydrogen-bonded polyacrylic acid (PAA) and polyvinylpyrrolidone (PVPON), increasing temperature, promotes hydrogen-bonding due to hydrophobic contributions, resulting in thicker bilayers [44]. Furthermore, referring to a previously mentioned polymer-pair, the temperature can be used to shrink PSS/PDADMAC capsules [44,49], as well as biopolymer analogues dextran sulphate/poly-L-arginine capsules [50]; this occurs due to the thermo-induced increase in mobility of these polymers, leading to the annealing (pore closure) of the PEM and subsequent capsule shrinkage (Figure 3(2)).

Although hydrogen bonds and electrostatic attractions can cooperate to immobilize a PEM particle on to a solid surface, they are mostly competing with adhesive, and capillary forces [44]. It is the ensemble of these interactions and forces that produces a theory for the immobilization of these PEM particles on to solid surfaces, and consequently, an insight into how future schemes can be improved upon. The strength of interaction and degree of immobilization can be controlled by adding metal, for example, gold nanoparticles onto the surface of polyelectrolyte multilayer films [5], where the distribution of gold nanoparticles is controlled by adsorption conditions (salts, ionic strengths, etc.) [51].

### 2.2. PEM Particle Morphology

As previously mentioned, shape influences the immobilization of particles onto solid surfaces. LbL PEM assemblies on spherical cores such as polystyrene (PS) (organic) or silica (SiO_2_) (inorganic) are most common, but cubic cores such as MnCO_3_ and CdCO_3_ (inorganic) have been also patterned [35,52,53,54].

In the case of SiO_2_ and MnCO_3_, the anisotropic nature of the latter poses complications in terms of its immobilization onto PS-patterned silicon oxide. Both particles have comparable dimensions (2.5 ± 0.2 μm), with the MnCO_3_ being cubic in nature whereas the SiO_2_ is spherical [55]. To confirm the LbL assembly of (PAH/PSS)_2_ shells on the cubic and spherical cores, ζ-potential measurements were taken (at pH 7 in an aqueous solution). Specifically, the ζ-potential is the difference in voltage, between the Stern layer and the diffuse layer [56]. It determines the energy required to bring two separate surfaces together, by considering their relative velocities [57]. During this review, it will be quoted when evaluating the surface charge of particles and surfaces [58].

**Figure 4:**
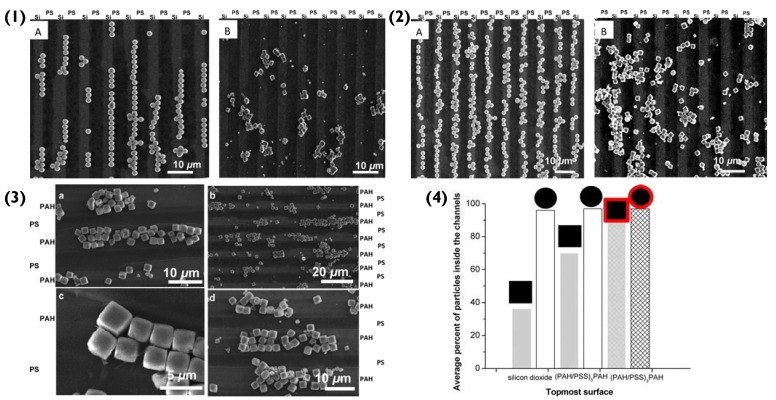
(**1**) Scanning electron microscopy (SEM) images of bare (**A**) spherical and (**B**) cubic microparticles on the PS-pattered silicon substrate. (**2**) SEM images of bare (**A**) spherical and (**B**) cubic microparticles on the pattered silicon substrate functionalised with a (PAH/PSS)_3.5_ coating. (**3**(**a**–**d**)) SEM images of (PAH/PSS)_2_ coated cubic microparticles assembled on the pattered silicon substrate functionalised with (PAH/PSS)_3.5_. (**4**) Average percentages of bare (empty columns) or (PAH/PSS)_2_ coated (cross-hatched columns) spherical and cubic microparticles on the patterned substrates. Reprinted with permission from reference [55] copyright © 2012 American Chemical Society.

The fluctuation of surface potentials was due to the deposition polycationic PAH (+40 mV) and polyanionic PSS (−35 mV) [59]. Prior to patterning, the manganese cores possessed a small negative surface charge, making the deposition of PAH feasible, which in turn changed the surface charge. This consequently enhanced the adhesion of the PSS electrostatically and via hydrogen bonds [60]. However, the immobilization of bare MnCO_3_ particles on to the solid surface (silicon wafer) was not preferentially controlled, as can be observed in Figure 4(1) Due to their large surface contact area (6.25 μm^2^), the cubic particles tether strongly on first impact because of adhesive forces, disregarding the composition of the immobilization site as they do not preferentially adhere to neither PS nor SiO on the silicon wafer [61]. In fact, the adhesive forces are four-times greater than those of the spherical SiO_2_ particles. The van der Waal forces involved would also increase in magnitude with increasing surface contact area. This removed the aspect of preferential immobilization. The adhesive forces of bare MnCO_3_ are so strong that they disregard the effect of the capillary force and result in random immobilization. Conversely, SiO_2_ particles strongly immobilize to the hydrophilic silicon substrate (95% successful immobilization), despite there being a repulsion between their negative charge and the one at the surface of the silicon oxide (solid platform). The immobilization of the spherical particles is predominantly controlled by capillary forces [62]. Compared to the cubic particles, the spherical particles have a small contact surface area (625 nm^2^), resulting in weak adhesive forces. Due to their small contact surface area, there is a smaller negative charge per unit area, meaning that the repelling effect between them and the silicon wafer is weaker than that of the MnCO_3_ particles and therefore not strong enough to impede their immobilization.

To establish semi-controlled immobilization, the silicon wafer was initially patterned via LbL coating. The outer layer of the solid surface was PAH, resulting in a +40 mV surface potential. This modification improved the immobilization of spherical particles to the positively charged areas by 3%, as aside from the existing capillary forces, there was now a strong electrostatic attraction aiding their adhesion. The immobilization of cubic particles on to the positively charged channels increased to 70%, showing a significant improvement (Figure 4(2)). However, even though the solid surface now had large positive surface potentials the ζ-potential on bare MnCO_3_ was weakly negative, meaning that a lot of cubic particles still flocculated. It must be noted at this stage that the PS on the substrate surface has a small negative potential. The resultant Coulombic interaction between the particles and the substrate however was four-orders of magnitude lower than the capillary forces present. The slight repulsion would be felt by all negatively charged particles and in the case of the spherical particles, may have guided their immobilization alongside the capillary force to the positively charged channels. However, in the case of the cubic particles, their adhesive forces were dominant enough to override this repulsion.

The particles were then functionalized via LbL assembly, with the outer shell being PSS. This resulted in more negative ζ-potentials and hence increased the immobilization probability (86%) of cubic microparticles on to the positively charged channels of the solid surface, as seen in Figure 4(3). Furthermore, the negative surface potential meant that the cubic particles were no longer immobilized randomly, as they now experienced a stronger electrostatic attraction to the patterned surface and displayed good stability in suspension [43]. Overall, the patterning of the cubic particles with a negatively charged shell resulted in a notable change in binding energy; PAH/(MnCO_3_)PSS compared to PAH/MnCO_3_. Specifically, the interaction energy (ΔW^tot^) between the modified surface and cubic particle was greater than the ΔW^tot^ between the PS regions and the anisotropic particles. Therefore, the final immobilization position of the cubic particles is a result of strong adhesive forces inside the channels and strong repulsive forces outside of them.

### 2.3. PEM Particle Agglomeration

As can be seen in Figure 4, cubic and spherical PEM particles form chain-like aggregates. On the one hand, cubic particles densely pack due to energetically favourable face-to-face interactions. On the other hand, spherical particles interact via a point-like contact area, which compared to the cubic particles is very small in size. Consequently, the interfacial hydrophobic energies between cubic particles are six-orders of magnitude greater than those between spherical particles [63]. The strength of the wall-to-wall interactions between cubic particles and their strong adhesive forces (due to the large contact surface between them and the substrate) is strong enough to compete with the capillary, van der Waals and Coulombic forces. Consequently, their agglomerate formation promotes further immobilization of PEM cubic particles to the solid surface and should be considered during application. The aggregation of these spherical and cubic particles on a solid surface is not directly influenced by LbL-assembled PEMs. However, since aggregation is linked to salt concentration, local microroughness, shell-thickness and shell-porosity, all of which are factors that are linked to the PEMs, it is fair to assume that particle aggregation is affected by the PEMs [64]. Synoptically, as the salt concentration increases, the percentage of aggregated particles per cluster increases. However, as mentioned previously, increased salt concentration can also affect shell porosity and can increase roughness [44]. The thickness of PEM shells also depends on the water content, as the shell thickness increases with swelling [35]; water content, however, is also dependent upon the polymers used [65]. Furthermore, the shape of the core can affect the roughness, thickness, and porosity of shells. This is because cubic particles stretch out the polymers on their surface more, because of their sharp corners, resulting in overall smoother surfaces, higher porosity, and thicker shells [35]. This will continue to be a paramount theme in this review; the theory for the immobilization of multi-layered particles on to solid surfaces is multifaceted and we must consider a plethora of factors and interactions in each scenario.

**Figure 5:**
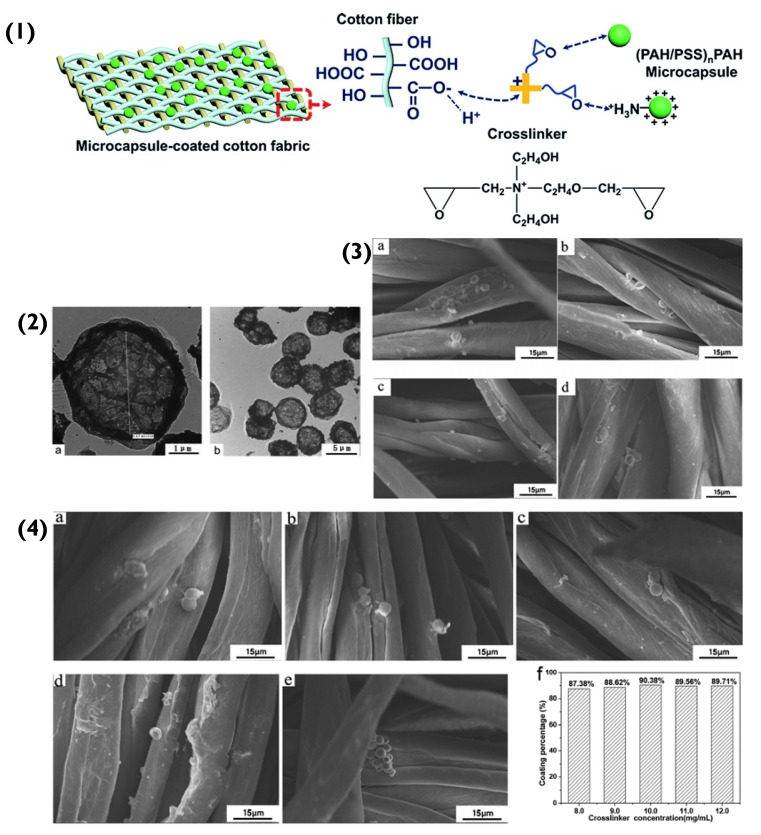
(**1**) Schematic representation of the binding of PEM capsules to the cotton fibres. (**2**(**a**,**b**)) Transmission electron microscopy images of (PAH/PSS)_2.5_ capsules. (**3**) SEM images of (PAH/PSS)_2.5_ microcapsule-coated cotton fabrics with different microcapsule numbers: (**a**) 3 *×* 10^7^; (**b**) 4 × 10^7^; (**c**) 5 × 10^7^; (**d**) 6 × 10^7^. (**4**) SEM images of (PAH/PSS)_2.5_ microcapsule-coated cotton fabric with different concentrations of crosslinker: (**a**) 8.0 mg/mL; (**b**) 9.0 mg/mL; (**c**) 10.0 mg/mL; (**d**) 11.0 mg/mL; (**e**) 12.0 mg/mL; (**f**) coating percentages microcapsules on cotton fabric at different concentrations of the crosslinker. Reprinted with permission from reference [69] copyright © 2020 Royal Society of Chemistry.

In an alternate study, it was reported that agglomeration hindered immobilization. Once again, LbL (PAH/PSS)_n_PAH particles were produced but, in this case, the initial core was PSS-doped vaterite CaCO_3_ crystals; capsules prepared were 4–6 μm in diameter, and imaged via electron microscopy (Figure 5). Compared to silica particles, CaCO_3_ cores require the use of mild, non-harmful conditions for their decomposition [66], and do not require dissolution agents such as HF, which can cause burns and tissue damage [67], as well as damage to the pre-encapsulated bioactive cargo. CaCO_3_ crystals are also completely decomposable and biocompatible and are reported to be able to retain the biological activity of the cargo encapsulated following its adsorption [21,68].

The immobilization of the (PAH/PSS)_n_PAH particles to a cotton fabric was initially achieved via electrostatic attraction (Schematic in Figure 5(1)). The positively charged amino group in PAH bonded to the negatively charged -COO^−^ (-COOH) in the cotton’s cellulose fibre. However, as the number of immobilized particles increased and agglomerated, the coating percentage on the fabric’s surface decreased. As mentioned previously, spherical particles have small interfacial hydrophobic energies between themselves because they bind (sphere-to-sphere) via point-like areas, that are small in magnitude. This means that the interparticle face-to-face interactions are on par with the Coulombic forces [64]. However, as more and more spherical particles aggregate (in this case, on the surface of the fabric), they begin to compete for an immobilization position and eventually due to increasing Coulombic forces, will repel each other. Unlike the cubic PEM particles mentioned previously, spherical particle aggregation appears to hinder the immobilization of other spherical particles. To overcome this immobilization repulsion factor, a crosslinker was introduced between PEM hollow-particles and the fabric. Essentially, the crosslinker contained ethylene oxide groups, which under neutral conditions interacted with the PAH’s NH_3_^+^ group via hydrogen bonds. The positively charged NR_4_^+^ of the crosslinker bonded electrostatically to the negatively charged -COO^−^ (-COOH) in the cotton cellulose fibre. This created a more favourable bonding system that overcame the repelling effect of aggregation and increased the successful immobilization percentage of hollow PEM particles from 87.38% to 90.38% (Figure 5(4)) [69].

## 3. PEM Particle Immobilization Strategy

### 3.1. Biopolymer-Based PEM Particle Immobilisation

**Figure 6:**
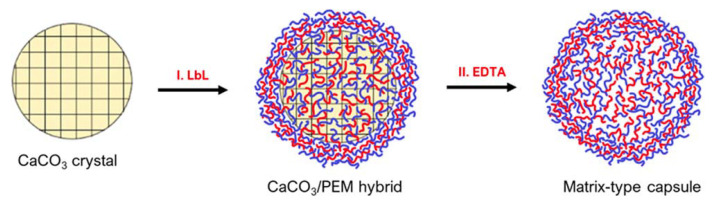
Schematic of PEM matrix-type capsule formation on vaterite calcium carbonate cores: (I) deposition of PEMs on CaCO_3_ crystals resulting in the permeation of the polymers inside the crystal pores and formation of the PEM shell on the crystal surface and (II) removal of CaCO_3_ crystals by the addition of a chelating Agent (EDTA), resulting in the formation of matrix-type capsules. Reprinted with permission from reference [3] copyright © 2021 American Chemical Society.

Matrix-type capsules are also common when using CaCO_3_ templates. These particles are formed from mesoporous CaCO_3_ cores being interpenetrated by polyelectrolytes during LbL deposition (Figure 6). For instance, confocal laser scanning microscopy (CLSM) displays that in vaterite CaCO_3_ crystal pores, the polymers that have interpenetrated, occupy the internal volume of the core homogenously [3]. Compared to hollow-particles, in the matrix-type, the polymers that were distributed inside the core remain post-dissolution, but instead of arranging in a shell formation, they remain in the particle’s lumen [3]. This lumen occupation becomes specifically important regarding the release of cargo from such capsules. Hollow-type capsules can resemble cells, as they provide a large, bordered inner space with superior mechanical and thermal stability that is separated from the outside environment [70].

**Figure 7:**
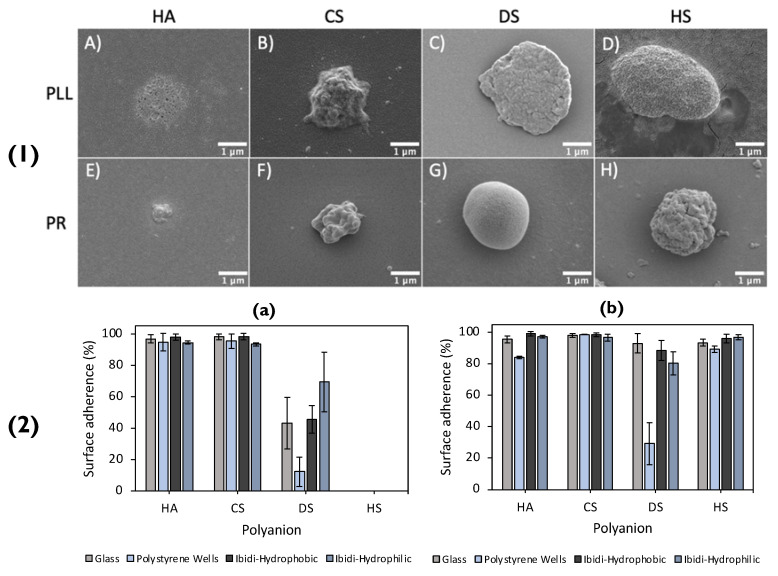
(**1**) SEM images of lyophilised (polyanion/polycation)_2.5_ microcapsules PLL-based (top row) capsules consisting of HA, CS, DS, and HS are shown in images (**A**), (**B**), (**C**) and (**D**), respectively. PR-based (bottom row) capsules, consisting of HA, CS, DS, and HS, are shown in images (**E**), (**F**), (**G**) and (**H**), respectively. (**2**) Adherence of PLL- (**a**) and PR- (**b**) based capsules upon different surfaces (specified within the legend). Reprinted with permission from reference [3] copyright © 2021 American Chemical Society.

Recently, a plethora of polymer-combinations was used to form matrix-type particles, that were then immobilized on to a glass surface [3]. The matrix-type particles were either poly-L-lysine (PLL)-based, or protamine (PR)-based and paired with either of the polyanions: HA, chondroitin sulphate (CS), dextran sulphate (DS) or heparin sulphate (HS), SEM images of which can be seen in Figure 7(1) Once the vaterite templates were dissolved via EDTA, the polymer matrices rearranged, reducing the size of the formed particles by up to factors of ~8 in diameter. The shrinkage of the particles was solely dependent on the polymer-pairs utilized. Synoptically, the particles based on PR displayed a higher degree of shrinkage compared to PLL. This was most likely due to the fact that highly charged PLL had a vast number of contact sites with the polyanions, resulting in slower chain dynamics during the dissolution of the core. There was an increasing shrinkage trend displayed for the equivalent polyanions in the PLL- or PR-based particles, following the series: DS ≈ HS < CS < HA. The purpose of referring to the degree of shrinkage in this scenario is because it directly correlates to the immobilization of these particles on the glass surface. In addition to this, the PR-based particles may have a large number of positive charges on their outer surface that are not compensated. This results in the strong electrostatic attraction between the PR-based particles and the glass surface, which is rich in negative silanol groups. On the other hand, even though PLL is polycationic, it has multiple contact points with the polyanions used, resulting in fewer available charged amino groups. This impedes the immobilization of PLL-based particles as the electrostatic attraction between them and the surface is weaker. Another contrast between the two types of particles was that the type of polyanion used impacted the immobilization of PLL-based particles, whereas it did not affect the PR-based particles. In detail, polyanions with more charged groups resulted in particles with lower immobilization percentages, because there were fewer available amino groups in the PLL (Figure 7(2)). This signifies the importance of the polymer charge density when immobilizing particles. The immobilization of the particles may have also been aided via hydrogen bonds, formed between the glass silanol groups and the amino groups present on the polycations.

In view of the successful adhesion on the glass surface, the same particles were immobilized on polystyrene and ibidi- hydrophobic/hydrophilic coated wells, displaying that the immobilization of these particles was not affected by altering the surface coating. Additionally, it further solidifies that the driving force behind the particle immobilization on to the glass substrate is their hydrophobic nature. With decreasing shrinkage coefficients, and hence decreasing water content with increased polyanion charge density, PLL-based particles displayed lower percentages of immobilization. On the contrary, PR-based particles demonstrated immobilization percentages close to 100% in all scenarios, and hence, did not display a relationship between immobilization and the degree of shrinkage and water content. The authors suggested this can most likely be attributed to PR’s globular structure, of which rearranges upon interaction with polyanions and immobilization upon the surface. Overall, these fully biopolymer-based PEM particles may be used for fully biocompatible coatings for future bioapplications (i.e., implant coatings).

**Figure 8:**
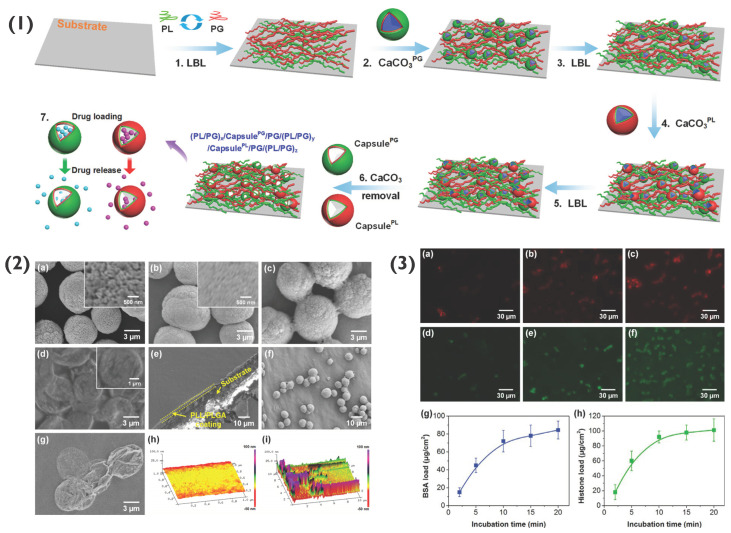
(**1**) Schematic demonstrating the formation of capsule-integrated PL/PG PEM films, where X^PG^ and X^PLL^ indicates PG and PLL-loaded particles, respectively. (**2**) SEM images of (**a**) CaCO_3_ particles, (**b**) CaCO_3_^PL^ particles, (**c**) cross-linked (PL/PG)_3.5_ coated CaCO_3_^PL^ particles, (**d**) capsule^PL^, (**e**) (PL/PG)_5_ PEM, (**f**) coated CaCO_3_ particles on (PL/PG)_5_ PEM, and (**g**) capsule^PL^ on (PL/PG)_5_ PEM. Atomic force microscopy (AFM) images of the PEM (**h**) without and (**i**) with capsules on capsule^PL^-integrated (PL/PG)_5_ PEM. (**3**) Confocal images of (PL/PG)_5_/capsule^PG^/(PL/PG)_8_/capsule^PL^/(PL/PG)_8_ PEMs loaded with BSA for (**a**) 2, (**b**) 10, and (**c**) 20 min, and loaded with histone for (**d**) 2, (**e**) 10 and (**f**) 20 min. The loading profiles of (**g**) BSA and (**h**) histone. Reprinted with permission from reference [77] copyright © American Chemical Society 2018.

Moreover, 2D polymer multilayers themselves may be used as hosts for PEM-coated or PEM particles [71]. Previously, such 2D PEM coatings have been used to host particles such as micelles [72], liposomes [73,74] and nanoparticles [32,75], for applications such as tissue engineering and biosensing [11,76]. PEMs formed of PLL and poly-L-glutamic acid (PLG) have been used to host both (PLL/PLG)_5_-coated CaCO_3_ particles as well as capsules for drug delivery applications. Here, both PLG- and PLL-loaded CaCO_3_ particles were used as components of the layer-by-layer assembly process (Figure 8), followed by exposure to EDTA to remove CaCO_3_, forming capsules. Following preparation, a range of deliverables including bovine serum albumin (BSA), silver nanoparticles, histone and rhBMP-2 were successfully encapsulated and released over a period of two weeks or longer [77]. Similar studies have been reported for the targeted delivery of interkeukin 12p70 and bone morphogenetic protein 2 [78]. Such coatings are possible to form due to the electrostatic interaction between the polymers within the PEM film as well as the final layer of the PEM particles, demonstrating the simple potential immobilisation of PEM particles within an array of polyelectrolyte films for various bio-applications, including antimicrobial or cell adhesive films, for instance.

Another type of biopolymer-based particle is encountered in mucoadhesion; a type of wet immobilization between a coated particle and a mucous tissue or membrane. These coated particles are ideal for mucosal drug delivery pathways as they: (1) protect the potential drug from digestive enzymes found in the gastrointestinal tract [79,80], (2) ensure its stability due to fluctuating pH levels and (3) are capable of mucopenetration in order to reach the epithelium.

The exterior of the mucus is negatively charged due to the presence of sialic acid inside mucins [81]. Furthermore, negatively charged glycoproteins found inside mucin allow for the formation of hydrogen bonds, due to strong proton donor/acceptor capabilities [81]. Therefore, to establish mucoadhesion, the outer layer of the particles must be able to bind electrostatically and/or via hydrogen bonds. An example of this is chitosan (CS) and sodium tripolyphosphate (TPP) nanoparticles that were tailored to immobilize electrostatically on the mucus surface, in order to deliver daptomycin for ocular treatment of bacterial endophthalmitis [82]. In addition to this, the surface chemistry of the particles could be modified further to allow adhesion to the mucus surface via disulfide bonding and van der Waals forces [83]. In the case of disulfide bridges, polymers can be thiolated so that they bond to the cysteine groups of mucin [84]. This was displayed in nanoparticles composed of maleimide-chitosan-catechol-alginate (Mal-CS-Cat-Alg) immobilized via the cysteine groups and bonded covalently with amines and thiols on the bladder mucus [85].

### 3.2. Surface Modification via Sol-Gels

Using the theory introduced thus far, a more complex immobilization pathway, involving patterned SiO_2_ particles on to an aluminium surface will now be discussed. Their adhesion to the solid surface is in the form of a film; a hybrid epoxy functionalised ZrO_2_/SiO_2_ and sol-gel.

**Figure 9:**
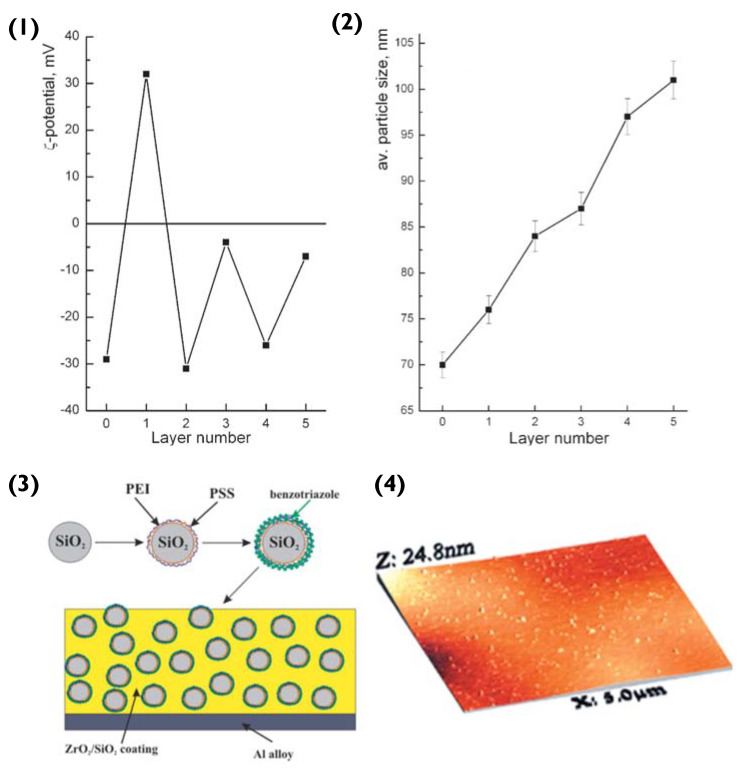
(**1**) Zeta potential and (**2**) size of nanoparticles in water during LbL assembly—Layer number 0: initial SiO_2_; 1: SiO_2_/PEI; 2: SiO_2_/PEI/PSS; 3: SiO_2_/PEI/PSS/benzotriazole; 4: SiO_2_/PEI/PSS/benzotriazole/PSS; and 5: SiO_2_/PEI/PSS/benzotriazole/PSS/benzotriazole. (**3**) Schematic illustration of the fabrication of the composite ZrO_2_/SiO_2_ coating loaded with benzotriazole nanoreservoirs and (**4**) Scanned topography of the sol-gel coating containing the LbL nanoreservoirs performed using AFM. Reprinted with permission from reference [86] copyright © 2006 John Wiley and Sons.

To begin with, polyethylene imine (PEI) and PSS layers are deposited onto the SiO_2_ particle, via LbL assembly. The surface ζ-potential of SiO_2_ is negative (−29 mV) [86]. Hence, the first deposited layer is that of the polycation PEI (resultant surface ζ-value of +36 mV), followed by the polyanion PSS (ζ −32 mV) [87]. The third layer added was that of the anticorrosion agent benzotriazole, resulting in a surface ζ-potential of −4 mV (Figure 9(1)). Compared to previous examples of PEM particles, the SiO_2_ does not encapsulate its cargo in a hollow-lumen or matrix. Instead, the benzotriazole is entrapped within the PEM during LbL assembly, to prevent it from interacting with the layer matrix. Here, we can refer to the theory introduced in Section 2.1. PEM layers are responsive to pH change, hence permitting this delivery system to release benzotriazole in response to corrosion induced pH change [88].

At this stage, it should be noted that compared to linear LbL assemblies, the benzotriazole should not be thought of as a complete outer layer, as it is interwoven into the PSS layer. The inhibitor immobilizes the PSS via electrostatic attraction and hydrogen bonding between the amino and sulfonate groups. Once the particles were prepared, they were embedded into hybrid ZrO_2_/SiO_2_ sol-gel films utilising zirconia- and organosiloxane-based sols, following the sol-gel procedure [86,87] (Figure 9(3)). These steps resulted in the creation of a film that was deposited on the aluminium alloy via dip-coating. This provided the alloy with a ‘self-healing’ coating. When corrosion commenced, a pH change was triggered, degrading the PSS/benzotriazole complex and hence releasing the anti-corrosion agent. The inhibitor formed a thin layer over the impaired metal surface and hindered further corrosion by replacing the damaged Al_2_O_3_ layer.

It would be expected from the theory introduced previously that the spherical particles would repel each other (Coulombic force) and due to their small surface contact area, this agglomeration would not be favoured. However, the authors mention the dense, homogeneity of the PEM particles within the sol-gel film. This was due to crosslinking agents that were added whilst the particles were still in solution to ensure that when the method got to the drying stage, the crosslinking agents chemically bonded with the particles to form crack-free coating [89].

Overall, in this scenario, the majority of the forces discussed in this review would still influence particle immobilization, but the effect would not be as dominant. Due to the sol-gel method, these particles would not experience the typical spatial freedom seen in the other examples of this review and would consequently be more confined. Coulombic forces between spherical particles would still try and prevent clustering, whilst the capillary force would influence the mobility of the particles before gelation, but the inclusion of cross-linkers solidifies the efficiency of this method.

Moreover, in a later study, particle PEMs were decorated with silver nanoparticles, allowing them to become both pH and laser-stimulated corrosion inhibitors [26]. This demonstrates the potential for such PEM particles as anti-corrosive materials in various coatings, including sol-gels and paints, for instance.

### 3.3. Polymer Brushes and Scaffolds with Incorporated Particles

Another and very different strategy of surface modification is based on grafting polymers on a substrate with subsequent polymerization. Functionalizing the surfaces with polymeric brushes is advantageous since it allows to modify and switch the surfaces with a very thin layer at the interface [90]. Responsiveness of polymeric brushes to different stimuli is another essential feature of such coatings; in this regard, incorporation of nanoparticles into polymeric brushes revealed a possibility of altering hydrophobicity/hydrophilicity, switchable mass transport, motions, reversible assembly disassembly of nanoparticles, etc. [91].

Immobilization of gold nanoparticles onto poly-(2-vinylpyridine) brushes has been shown to lead enhance plasmonic effect [92]. Recently, non-fouling poly(di(ethylene glycol)methyl ether methacrylate) brushes were functionalized with calcium carbonate particles [93], which can be used to control cell adhesion to such surfaces. In fact, similar conclusions were drawn from studying cell adhesion on cell non-friendly hydrogel coatings, where surface-incorporated calcium carbonate particles were shown to serve as adhesion centers [94].

In addition, particles have been functionalized with brushes. For example, silica particles were incorporated and extensively characterized [95]. Subsequently, silica particles functionalized with brushes were used for drug delivery [96]. Additionally, quantum dots were shown to be functionalized with brushes leading to vesicle formation [97].

Further, other matrices such as polycaprolactone scaffolds were functionalized with calcium carbonate particles, which were shown to stimulate vascularization in vivo [98].

### 3.4. Surface Patterning and Microcapsule Arrays

There are several patterning techniques that allow the positioning nano- and microparticles onto solid surfaces. In general, surface patterning is represented by top-down micro- and nanolithographic techniques and bottom-up chemical methods, including self-assembly of micro- and nano-structure [99,100]. While lithography allows the fabrication of patterned thin films with desired geometries and dimensions, bottom-up approaches enable the positioning of nano- and microparticles (pre-fabricated or self-assembled directly on the surface) onto solid surfaces. Depending on the scale, surface patterning techniques belong either to the methods of microfabrication, if the processes can reliably produce features of microscopic size such as ten micrometres or less; or nanofabrication, if the processes can produce nanoscale features, such as less than 100 nanometres.

Microlithography is classically used in the semiconductor industry and also for the manufacture of microelectromechanical systems. In recent decades, it also found its niche in Biomaterials, for their manufacture at small scales [100]. Nowadays, neither self-assembly nor top-down lithographic approaches can adequately fulfil all the requirements for surface patterning. Lithographic technologies represent a powerful apparatus for surface modification, providing high uniformity and consistency in terms of controlling specific size, shape, and functionality of the patterns. This is in contrast to the self-assembled patterns, which often exhibit dynamic instability and therefore present challenges in manipulation of exact size, shape, and encapsulated drug doses [100]. The assembly of defect-free arrays with true long-range order remains challenging. From the other side, nanolithography has not yet demonstrated success in constructing less then tens of nanometer patterns. Although significant progress has been made in terms of nanolithography even at smaller length-scales [101,102], such advances are accompanied by the technological barriers and the use of unique state-of-the-art instrumentation [99,102]. Besides, the lithographic approach typically yields primitive planar geometries and lacks architectural diversity, while self-assembly allows the formation of morphologically and compositionally sophisticated structures. The latter is especially important for biomedical applications. Recent reports also more and more often relied on a hybrid strategy that utilizes lithographically defined masks to direct the self-assembly [99].

There are a few studies where lithographic techniques met LbL technology. Thus, microporous polymer nanofilms were grown in a sequential LbL manner and patterned by photo lithography during their growth using the photomask [103]. In [104], polymer capsules were deposited on a solid support and the surface was then patterned by electron beam lithography. Electron beam irradiation resulted in the increase of the capsule adhesion to the surface, while the capsules in the non-irradiated areas were washed out. Most often, when the PEMs are assembled with the assistance of lithographic techniques, this is about soft lithography [105,106].

**Figure 10:**
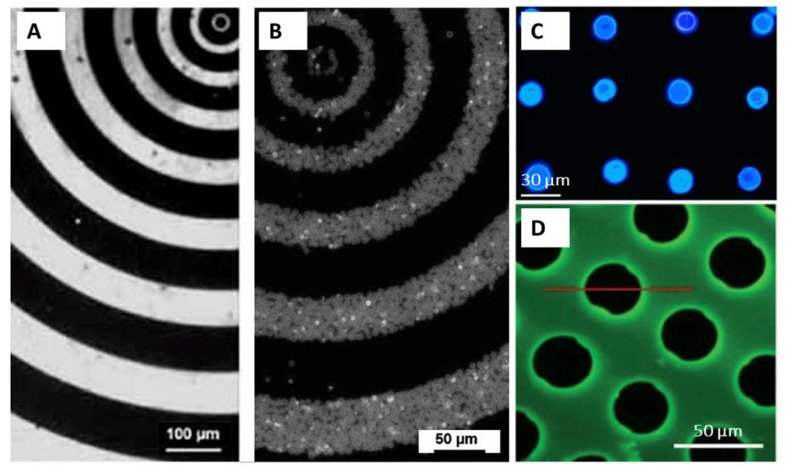
(**A**,**B**)—Fluorescence microscopy images of FITC-PAH pattern created on glass substrates (**A**) and corresponding PSS/FITC-PAH microcapsule array (**B**). With permission from [111]. (**C**) Fluorescence microscopy image showing the existence of ZnS quantum dots (blue) formed exclusively within the patterned microcapsules patterned on PET film. With permission from [112]. (**D**) Fluorescence microscopy image of HA/PLL multilayer film obtained after the LbL microfluidics-assisted deposition and before the disassembly of the glass substrate from the PDMS mask.

In most of the studies, PEMs are self-assembled on the surfaces without any pattern, forming homogeneous thin coatings [107]. Actually, it is of note that the widely accepted assertion about the smoothness of the LbL films is not always true: the LbL coatings could also be rough and even spontaneously form patterns during film build-up under certain conditions [108,109]. Using the micro-structured silicon rubber mould [105] and microfluidics-assisted growth [110] of the LbL films, controlled fabrication of micro-patterned PEMs can be achieved. For instance, HA/PLL films were grown in 3D with the pattern illustrated in Figure 10, D providing advanced support for selective cell growth [110]. However, patterning at smaller length-scales using these approaches remains impossible. This challenge may be avoided if using the immobilization of prefabricated nano- and micro-particles made of PEMs instead of PEM fabrication on the surface. Another advantage of immobilization is that it provides an intriguing opportunity to mix the particles of various compositions (e.g., microcapsules hosting various functional payloads), providing extremely high diversity in tailoring surface properties. In this respect, patterned immobilization of nano- and micro-particles made of PEMs might become a powerful surface modification, if control over the immobilization is achieved.

For instance, semi-controlled immobilization of the microparticles on a LbL-coated silicon wafer is illustrated in Figure 4 and discussed in the corresponding section. An example of the patterning of PEM microcapsules with electron-beam irradiation has also been mentioned above. More commonly, electrostatic coupling of the microcapsules to the surface is employed. Thus, glutaraldehyde-cross-linked PSS/PAH microcapsules templated on 4–6 µm vaterite crystals were supported onto the surface pre-treated with PAH [111]. The microcapsules reacted with the amino groups on the surface via free aldehyde groups on their outer layer (Schiff base –CH=N– linkages). A representative example of the mask and corresponding final capsule pattern is shown in Figure 10A,B. In [112], biotinylated PSS/PAH microcapsules templated on ca. 15 µm vaterite crystals were immobilised onto avidin patterns on a poly(ethylene terephthalate) (PET) film (Figure 10C). The immobilisation was driven by biotin-avidin affinity.

The particles described above were fabricated using the LbL assembly of polymers onto solid substrates with or without the removal of the substrate. Structures that can be assembled using the LbL polymer coating may also include free-standing multicomponent or one-component structures made the same way using decomposable cores such as protein aggregates [113,114] or vaterite crystals [115]. Plenty of other structures can be obtained such as pure protein particles using the LbL assembly and hard templating on vaterite crystals [116]. This brings almost no limit to a variety of structures for immobilization onto solid surfaces and huge opportunities for further research and applications (also biologically related) in this filed.

## 4. Confirming Immobilization

The final stage of PEM particle immobilization involves the confirmation of this process using surface analysis. In general, this is accomplished with the aid of SEM. This imaging method irradiates the sample on which the PEM particles are immobilized with a low-energy electron beam. Synoptically, the beam interacts with the irradiated surface (either particle or solid platform) and results in the emission of photons and electrons [117]. Once the secondary electrons scatter from the source, they are ‘picked-up’ by the detector, which in turn will transmit the signals to a computer, that will process them and produce a 3D image. Samples that do not contain conductive materials are usually sputter-coated, to increase the signal/noise ratio and improve image definition (Figure 7(1)).

In another study [38], CLSM was used to identify the PEM particles (Figure 2(4)). For this method, the particles were loaded with FITC. This was done by adding FITC dropwise to the polyelectrolyte solutions prior to the LbL assembly. In detail, microscopy uses a pinhole to direct filtered light at the sample. The light that passes through the pinhole has the appropriate wavelength to excite the FITC in the particles, which in turn emit fluorescent light of another wavelength. This returning light passes through a dichroic mirror and filter before reaching the detector. The incoming light is then converted to a signal and finally, a 2D image is produced. Compared to SEM, CLSM can help characterize the internal structure of the PEM particles in a wet state and in real-time that is critically important to investigate the original structure (SEM can only analyse dried samples).

Another method used for confirming immobilization is AFM. A cantilever is irradiated with a laser whilst its tip brushes the surface of the solid platform. Variations in the topography result in the jolting of the sensitive cantilever tip, hence altering the beam picked up by the photodiode. This type of microscopy was used to confirm the immobilization of the anticorrosion particles in Figure 9. Compared to SEM, AFM does not require pre-treatment of the sample and the sample can be in an either dry or wet state. However, AFM cannot perform scans as fast as SEM imaging and can only analyse rather small features (typically below 10 microns in height).

Table 1 below summarises the findings of this review focusing on particle type and shape, polymers used, surface immobilization method and analysis techniques as well as forces responsible for the immobilization.

## 5. Conclusions

This review describes approaches and mechanisms for the immobilization of LbL-assembled PEM particles (capsules and PEM-coated particles) on to solid surfaces. The forces (Coulombic, capillary, adhesive, etc.) and factors (shape, clustering, charge, pH, and salt concentration) that govern the immobilization were discussed. The investigation displayed numerous immobilization examples, each contributing new knowledge that complimented and built upon the previously discussed theory, including the complex immobilization mechanism for PEM particle-integrated sol-gel coatings. Approaches for the confirmation of particle immobilisation are also highlighted, including electron microscopies, CLSM and atomic force microscopy to both investigate and explore surface properties and topographies.

Each PEM particle example discussed here displayed its unique properties and applications in an array of different scientific fields, such as surface coatings (e.g., anti-corrosive, cell adhesive, biosensors, etc.), cargo carriers for drug delivery and physical applications, as well as material enhancement. The polyelectrolytes utilised for such particles can be biocompatible and biodegradable, making them an invaluable asset in the pursuit for greener alternatives in chemical research.

The multitude of tuneable functions and the low cost of the LbL-assembled particles make them an accessible tool to all. Their component availability and biocompatibility will see further innovation in their immobilization mechanisms and (bio)applications, stimulating progress and revolutionizing this field of materials science. Overall, there is relatively little literature exploring this field, and we believe such PEM particle-based coatings will succeed as tools for the formulation of novel, bespoke, yet simple materials for a variety of (bio)applications.

## Figures and Tables

**Table 1:** Synopsis of the main immobilization examples covered in this review.

Particle Core	Shape	Polymers Coated (Abbreviated)	Surface of Immobilization	Forces/Factors Influencing Immobilization	Technique for Immobilization Confirmation	Refs
MnCO_3_	Cubic	(PAH/PSS)	PS-pattered silicon substrate	Adhesive forces and agglomeration. Coulombic repulsion outside of the channels ensured anchoring of particles.	SEM	[55]
SiO_2_	Spherical	(PAH/PSS)	PS-pattered silicon substrate	Capillary force and electrostatic attraction in the channels. Coulombic repulsion outside of the channels ensured anchoring of particles.	SEM	[55]
CaCO_3_	Spherical	(PAH/PSS)_n_PAH	Cotton Fibres	Electrostatic attraction and hydrogen bonding via the usage of a cross-linker	SEM	[69]
CaCO_3_	Spherical	(HA/PLL)	Glass surface	Electrostatic attraction and hydrophobic interactions.	SEM/CLSM	[3]
CaCO_3_	Spherical	(HA/PR)	Glass surface	Electrostatic attraction and hydrophobic interactions.	SEM/CLSM	[3]
CaCO_3_	Spherical	(CS/PLL)	Glass surface	Electrostatic attraction and hydrophobic interactions.	SEM/CLSM	[3]
CaCO_3_	Spherical	(CS/PR)	Glass surface	Electrostatic attraction and hydrophobic interactions.	SEM/CLSM	[3]
CaCO_3_	Spherical	(DS/PLL)	Glass surface	Electrostatic attraction and hydrophobic interactions.	SEM/CLSM	[3]
CaCO_3_	Spherical	(DS/PR)	Glass surface	Electrostatic attraction and hydrophobic interactions.	SEM/CLSM	[3]
CaCO_3_	Spherical	(HS/PLL)	Glass surface	Electrostatic attraction and hydrophobic interactions.	SEM/CLSM	[3]
CaCO_3_	Spherical	(HS/PR)	Glass surface	Electrostatic attraction and hydrophobic interactions.	SEM/CLSM	[3]
CaCO_3_	Spherical	(PL/PG)	Polypeptide films	Electrostatic attraction between PEM films and final layer of PEM particles.	SEM/CLSM/AFM	[78]
Na_5_P_3_O_10_	Spherical	(CS)	Ocular mucosa	Electrostatic attraction and hydrogen bonding.	SEM	[82]
N/A	Spherical	(Mal-CS-Cat-Alg)	Bladder mucosa	Disulfide bridges and covalent bonding.	TEM, CLSM	[85]
SiO_2_	Spherical	(PEI/PSS/Benzotriazole)	ZrO_2_/SiO_2_ sol gel	Coulombic forces, capillary forces and cross-linkers.	AFM	[86]
CaCO_3_	Spherical	(PSS/PAH)	Glass surface treated with PAH	Schiff base –CH=N– linkages	CLSM	[104]

## Data Availability

Not applicable.

## References

[1] Richardson J.J., Cui J., Bjornmalm M., Braunger J.A., Ejima H., Caruso F. (2016). Innovation in Layer-by-Layer Assembly. Chem. Rev..

[2] Schuetz P., Caruso F. (2004). Semiconductor and Metal Nanoparticle Formation on Polymer Spheres Coated with Weak Polyelectrolyte Multilayers. Chem. Mater..

[3] Campbell J., Abnett J., Kastania G., Volodkin D., Vikulina A.S. (2021). Which Biopolymers Are Better for the Fabrication of Multilayer Capsules? A Comparative Study Using Vaterite CaCO_3_ as Templates. ACS Appl. Mater. Interfaces.

[4] Del Mercato L.L., Rivera-Gil P., Abbasi A.Z., Ochs M., Ganas C., Zins I., Sonnichsen C., Parak W.J. (2010). LbL multilayer capsules: Recent progress and future outlook for their use in life sciences. Nanoscale.

[5] Kohler D., Madaboosi N., Delcea M., Schmidt S., De Geest B.G., Volodkin D.V., Mohwald H., Skirtach A.G. (2012). Patchiness of Embedded Particles and Film Stiffness Control through Concentration of Gold Nanoparticles. Adv. Mater..

[6] Skorb E.V., Mohwald H. (2014). “Smart” Surface Capsules for Delivery Devices. Adv. Mater. Interfaces.

[7] Volodkin D.V., Delcea M., Mohwald H., Skirtach A.G. (2009). Remote Near-IR Light Activation of a Hyaluronic Acid/Poly(l-lysine) Multilayered Film and Film-Entrapped Microcapsules. ACS Appl. Mater. Interfaces.

[8] Tovani C.B., Faria A.N., Ciancaglini P., Ramos A.P. (2019). Collagen-supported CaCO_3_ cylindrical particles enhance Ti bioactivity. Surf. Coat. Technol..

[9] Zhao S., Caruso F., Dahne L., Decher G., De Geest B.G., Fan J., Feliu N., Gogotsi Y., Hammond P.T., Hersam M.C. (2019). The Future of Layer-by-Layer Assembly: A Tribute to ACS Nano Associate Editor Helmuth Mohwald. ACS Nano.

[10] Tu W., Zhou Y., Liu Q., Tian Z., Gao J., Chen X., Zhang H., Liu J., Zou Z. (2012). Robust Hollow Spheres Consisting of Alternating Titania Nanosheets and Graphene Nanosheets with High Photocatalytic Activity for CO_2_ Conversion into Renewable Fuels. Adv. Funct. Mater..

[11] Sato K., Takahashi S., Anzai J. (2012). Layer-by-layer Thin Films and Microcapsules for Biosensors and Controlled Release. Anal. Sci..

[12] Kida T., Mouri M., Akashi M. (2006). Fabrication of hollow capsules composed of poly(methyl methacrylate) stereocomplex films. Angew. Chem. Int. Ed..

[13] Wang Z.P., Feng Z.Q., Gao C.Y. (2008). Stepwise assembly of the same polyelectrolytes using host–guest interaction to obtain microcapsules with multiresponsive properties. Chem. Mater..

[14] Johnston A.P.R., Read E.S., Caruso F. (2005). A Molecular Beacon Approach to Measuring the DNA Permeability of Thin Films. Nano Lett..

[15] Cohen-Stuart M.A., Huck W.T.S., Genzer J., Muller M., Ober C., Stamm M., Sukhorukov G.B., Szleifer I., Tsukruk V.V., Urban M. (2010). Emerging applications of stimuli-responsive polymer materials. Nat. Mater.

[16] Shipway A.N., Caruso F. (2004). Small is beautiful. Chem. Phys. Chem..

[17] Parakhonskiy B., Yashchenok A.M., Mohwald H., Volodkin D., Skirtach A.G. (2017). Release from Polyelectrolyte Multilayer Capsules in Solution and on Polymeric Surfaces. Adv. Mater. Interfaces.

[18] Burmistrov I.A., Veselov M.M., Mikheev A.V., Borodina T.N., Bukreeva T.V., Chuev M.A., Starchikov S.S., Lyubutin I.S., Artemov V.V., Khmelenin D.N. (2022). Permeability of the Composite Magnetic Microcapsules Triggered by a Non-Heating Low-Frequency Magnetic Field. Pharmaceutics.

[19] Kalenichenko D., Nifontova G., Karaulov A., Sukhanova A., Nabiev I. (2021). Designing Functionalized Polyelectrolyte Microcapsules for Cancer Treatment. Nanomaterials.

[20] Trushina D.B., Bukreeva T.V., Antipina M.N. (2016). Size-Controlled Synthesis of Vaterite Calcium Carbonate by the Mixing Method: Aiming for Nanosized Particles. Cryst. Growth Des..

[21] Vikulina A., Webster J., Voronin D., Ivanov E., Fakhrullin R., Vinokurov V., Volodkin D. (2021). Mesoporous additive-free vaterite CaCO_3_ crystals of untypical sizes: From submicron to Giant. Mater. Des..

[22] Tong W., Song X., Gao C. (2012). Layer-by-layer assembly of microcapsules and their biomedical applications. Chem. Soc. Rev..

[23] Donath E., Sukhorukov G.B., Caruso F., Davis S.A., Mohwald H. (1998). Novel Hollow Polymer Shells by Colloid-Templated Assembly of Polyelectrolytes. Angew. Chem. Int. Ed..

[24] Shchepelina O., Kozlovskaya V., Kharlampieva E., Mao W., Alexeev A., Tsukruk V.V. (2010). Anisotropic Micro- and Nano-Capsules. Macromol. Rapid Commun..

[25] Kidambi S., Dai J., Li J., Bruening M.L. (2004). Selective Hydrogenation by Pd Nanoparticles Embedded in Polyelectrolyte Multilayers. J. Am. Chem. Soc..

[26] Skorb E.V., Skirtach A.G., Sviridov D.V., Shchukin D.G., Mohwald H. (2009). Laser-Controllable Coatings for Corrosion Protection. ACS Nano.

[27] Popova N.R., Popov A.L., Ermakov A.M., Reukov V.V., Ivanov V.K. (2020). Ceria-Containing Hybrid Multilayered Microcapsules for Enhanced Cellular Internalisation with High Radioprotection Efficiency. Molecules.

[28] Kozlovskaya V., Alford A., Dolmat M., Ducharme M., Caviedes R., Radford L., Lapi S.E., Kharlampieva E. (2020). Multilayer Microcapsules with Shell-Chelated 89Zr for PET Imaging and Controlled Delivery. ACS Appl. Mater. Interfaces.

[29] Wang Y., Hosta-Rigau L., Lomas H., Caruso F. (2011). Nanostructured polymer assemblies formed at interfaces: Applications from immobilization and encapsulation to stimuli-responsive release. Chem. Phys. Chem..

[30] Skirtach A.G., Yashchenok A.M., Mohwald H. (2011). Encapsulation, release and applications of LbL polyelectrolyte multilayer capsules. Chem. Commun..

[31] Kim S., Park J., Cho J. (2010). Layer-by-layer assembled multilayers using catalase-encapsulated gold nanoparticles. Nanotechnology.

[32] Lengert E., Koltsov S.I., Li J., Ermakov A.V., Parakhonskiy B., Skorb E.V., Skirtach A. (2020). Nanoparticles in Polyelectrolyte Multilayer Layer-by-Layer (LbL) Films and Capsules—Key Enabling Components of Hybrid Coatings. Coatings.

[33] Sergeeva A.S., Gorin D.A., Volodkin D.V. (2013). Polyelectrolyte Microcapsule Arrays: Preparation and Biomedical Applications. BioNanoScience.

[34] Feoktistova N., Rose J., Prokopovic V.Z., Vikulina A.S., Skirtach A., Volodkin D. (2016). Controlling the Vaterite CaCO_3_ Crystal Pores. Design of Tailor-Made Polymer Based Microcapsules by Hard Templating. Langmuir.

[35] Shchepelina O., Lisunova M.O., Drachuk I., Tsukruk V.V. (2012). Morphology and Properties of Microcapsules with Different Core Releases. Chem. Mater..

[36] Wohl B.M., Engbersen J.F. (2012). Responsive layer-by-layer materials for drug delivery. J. Control. Release.

[37] Mauser T., Dejugnat C., Sukhorukov G.B. (2004). Reversible pH-Dependent Properties of Multilayer Microcapsules Made of Weak Polyelectrolytes. Macromol. Rapid Commun..

[38] Szarpak A., Cui D., Dubreuil F., De Geest B.G., De Cock L.J., Picart C., Auzély-Velty R. (2010). Designing Hyaluronic Acid-Based Layer-by-Layer Capsules as a Carrier for Intracellular Drug Delivery. Biomacromolecules.

[39] Kempe K., Noi K.F., Ng S.L., Müllner M., Caruso F. (2014). Multilayered polymer capsules with switchable permeability. Polymer.

[40] Bucatariu F., Ghiorghita C.A., Dragan E.S. (2018). Cross-linked multilayer films deposited onto silica microparticles with tunable selectivity for anionic dyes. Colloids Surf. A.

[41] Becker A.L., Zelikin A.N., Johnston A.P.R., Caruso F. (2009). Tuning the Formation and Degradation of Layer-by-Layer Assembled Polymer Hydrogel Microcapsules. Langmuir.

[42] Zelikin A.N., Quinn J.F., Caruso F. (2006). Disulfide cross-linked polymer capsules: En route to biodeconstructible systems. Biomacromolecules.

[43] Kozlovskaya V., Kharlampieva E., Drachuk I., Cheng D., Tsukruk V.V. (2010). Responsive microcapsule reactors based on hydrogen-bonded tannic acid layer-by-layer assemblies. Soft Matter.

[44] Kharlampieva E., Sukhishvili S.A. (2006). Hydrogen-Bonded Layer-by-Layer Polymer Films. Macromol. Sci..

[45] Ghiorghita C., Dragan E.S. (2020). Polyelectrolyte Multilayer Thin Films Assembled Using Poly(N,N-dimethylaminoethyl methacrylate) and Polysaccharides: Versatile Platforms towards Protein Immobilization, Sorption of Organic Pollutants and Synthesis of Silver Nanoparticles. Proceedings.

[46] Köhler K., Biesheuvel P.M., Weinkamer R., Möhwald H., Sukhorukov G.B. (2006). Salt-Induced Swelling-to-Shrinking Transition in Polyelectrolyte Multilayer Capsules. Phys. Rev. Lett..

[47] Lisunova M.O., Drachuk I., Shchepelina O.A., Anderson K.D., Tsukruk V.V. (2011). Direct Probing of Micromechanical Properties of Hydrogen-Bonded Layer-by-Layer Microcapsule Shells with Different Chemical Compositions. Langmuir.

[48] Riegler H., Essler F. (2002). Polyelectrolytes. 2. Intrinsic or Extrinsic ChargeCompensation? Quantitative Charge Analysis of PAH/PSSMultilayers. Langmuir.

[49] Van der Meeren L., Li J., Konrad M., Skirtach A.G., Volodkin D., Parakhonskiy B.V. (2020). Temperature Window for Encapsulation of an Enzyme into Thermally Shrunk, CaCO_3_ Templated Polyelectrolyte Multilayer Capsules. Macromol. Biosci..

[50] Trushina D.B., Bukreeva T.V., Borodina T.N., Belova D.D., Belyakov S., Antipina M.N. (2018). Heat-driven size reduction of biodegradable polyelectrolyte multilayer hollow capsules assembled on CaCO_3_ template. Colloids Surf. B Biointerfaces.

[51] Skirtach A.G., Volodkin D.V., Möhwald H. (2010). Bio-interfaces—Interaction of PLL/HA Thick Films with Nanoparticles and Microcapsules. ChemPhysChem.

[52] Angelatos A.S., Katagiri K., Caruso F. (2006). Bioinspired colloidal systems vialayer-by-layer assembly. Soft Matter.

[53] Tang Z., Zhang Z., Wang Y., Glotzer S.C., Kotov N.A. (2006). Self-Assembly of CdTe Nanocrystals into Free-Floating Sheets. Science.

[54] Holt B., Lam R., Meldrum F.C., Stoyanov S.D., Paunov V.N. (2007). Anisotropic nano-papier mache microcapsules. Soft Matter.

[55] Lisunova M., Holland N., Shchepelina O., Tsukruk V.V. (2012). Template-Assisted Assembly of the Functionalized Cubic and Spherical Microparticles. Langmuir.

[56] Olthuis W., Schippers B., Eijkel J., van den Berg A. (2005). Energy from streaming current and potential. Sens. Actuators B.

[57] Ghernaout D., Al-Ghonamy A.I., Naceur M.W., Boucherit A., Messaoudene N.A., Aichouni M., Mahjoubi A.A., Elboughdiri N.A. (2015). Controlling Coagulation Process: From Zeta Potential to Streaming Potential. Am. J. Environ. Prot..

[58] Delgado A.V., González-Caballero F., Hunter R.J., Koopal L.K., Lyklema J. (2007). Measurement and interpretation of electrokinetic phenomena. J. Colloid Interface Sci..

[59] Zhu H., Stein E.W., Lu Z., Lvov Y.M., McShane M.J. (2005). Synthesis of Size-Controlled Monodisperse Manganese Carbonate Microparticles as Templates for Uniform Polyelectrolyte Microcapsule Formation. Chem. Mater..

[60] Kozlovskaya V., Higgins W., Chen J., Kharlampieva E. (2011). Shape switching of hollow layer-by-layer hydrogel microcontainers. Chem. Commun..

[61] Tsukruk V.V., Singamaneni S. (2012). Scanning Probe Microscopy of Soft Matter: Fundamentals and Practices.

[62] Schweikart A., Pazos-Perez N., Alvarez-Puebla R.A., Fery A. (2011). Controlling inter-nanoparticle coupling by wrinkle-assisted assembly. Soft Matter.

[63] Lisunova M.O. (2019). Assembly Controlled by Shape. MRS Adv..

[64] Lisunova M., Dorokhin A., Holland N., Shevchenko V.V., Tsukruk V.V. (2013). Assembly of the anisotropic microcapsules in aqueous dispersions. Soft Matter.

[65] Crouzier T., Picart C. (2009). Ion Pairing and Hydration in Polyelectrolyte Multilayer Films Containing Polysaccharides. Biomacromolecules.

[66] Vikulina A.S., Campbell J. (2021). Biopolymer-Based Multilayer Capsules and Beads Made via Templating: Advantages, Hurdles and Perspectives. Nanomaterials.

[67] Gnanadhas D.P., Thomas B., Elango M., Raichur A.M., Chakravortty D. (2013). Chitosan-dextran sulphate nanocapsule drug delivery system as an effective therapeutic against intraphagosomal pathogen Salmonella. J. Antimicrob. Chemother..

[68] Feoktistova N.A., Vikulina A.S., Balabushevich N.G., Skirtach A.G., Volodkin D. (2020). Bioactivity of catalase loaded into vaterite CaCO_3_ crystals via adsorption and co-synthesis. Mater. Des..

[69] Zhao Z., Li Q., Gong J., Li Z., Zhang J. (2020). A poly(allylamine hydrochloride)/poly(styrene sulfonate) microcapsule-coated cotton fabric for stimulus-responsive textiles. RSC Adv..

[70] Hong J., Han J.Y., Yoon H., Joo P., Lee T., Seo E., Char K., Kim B.S. (2011). Carbon-based layer-by-layer nanostructures: From films to hollow capsules. Nanoscale.

[71] Volodkin D., Skirtach A., Möhwald H. (2010). LbL Films as Reservoirs for Bioactive Molecules. Bioact. Surf..

[72] Cai H., Wang P., Zhang D. (2019). pH-responsive linkages-enabled layer-by-layer assembled antibacterial and antiadhesive multilayer films with polyelectrolyte nanocapsules as biocide delivery vehicles. J. Drug Deliv. Sci. Technol..

[73] Volodkin D.V., Schaaf P., Möhwald H., Voegel J.C., Ball V. (2009). Effective embedding of liposomes into polyelectrolyte multilayered films: The relative importance of lipid-polyelectrolyte and interpolyelectrolyte interactions. Soft Matter.

[74] Tang J.S.J., Smaczniak A.D., Tepper L., Rosencrantz S., Aleksanyan M., Dähne L., Rosencrantz R.R. (2022). Glycopolymer Based LbL Multilayer Thin Films with Embedded Liposomes. Macromol. Biosci..

[75] Schmidt S., Madaboosi N., Uhlig K., Köhler D., Skirtach A., Duschl C., Möhwald H., Volodkin D.V. (2012). Control of Cell Adhesion by Mechanical Reinforcement of Soft Polyelectrolyte Films with Nanoparticles. Langmuir.

[76] Kastania G., Campbell J., Mitford J., Volodkin D. (2020). Polyelectrolyte Multilayer Capsule (PEMC)-Based Scaffolds for Tissue Engineering. Micromachines.

[77] Zhang S., Xing M., Li B. (2018). Capsule-Integrated Polypeptide Multilayer Films for Effective pH-Responsive Multiple Drug Co-Delivery. ACS Appl. Mater. Interfaces.

[78] Zhang S., Vaida J., Parenti J., Lindsey B.A., Xing M., Li B. (2021). Programmed Multidrug Delivery Based on Bio-Inspired Capsule-Integrated Nanocoatings for Infected Bone Defect Treatment. ACS Appl. Mater. Interfaces.

[79] Sheng J., He H., Han L., Qin J., Chen S., Ru G., Yang V.C. (2016). Enhancing insulin oral absorption by using mucoadhesive nanoparticles loaded with LMWP-linked insulin conjugates. J. Control. Release.

[80] Sadeghi S., Lee W.K., Kong S.N., Shetty A., Drum C.L. (2020). Oral administration of protein nanoparticles: An emerging route to disease treatment. Pharmacol. Res..

[81] Bayer I.S. (2022). Recent Advances in Mucoadhesive Interface Materials, Mucoadhesion Characterization, and Technologies. Adv. Mater. Interfaces.

[82] Silva N.C., Silva S., Sarmento B., Pintado M. (2015). Chitosan nanoparticles for daptomycin delivery in ocular treatment of bacterial endophthalmitis. Drug Deliv..

[83] Sosnik A., Neves J.-D., Sarmento B. (2014). Mucoadhesive polymers in the design of nano-drug delivery systems for administration by non-parenteral routes: A review. Int. Sch. Res. Not..

[84] Sharma R., Ahuja M. (2011). Thiolated pectin: Synthesis, characterization and evaluation as a mucoadhesive polymer. Carbohydr. Polym..

[85] Sahatsapan N., Rojanarata T., Ngawhirunpat T., Opanasopit P., Patrojanasophon P. (2021). Doxorubicin-loaded chitosan-alginate nanoparticles with dual mucoadhesive functionalities for intravesical chemotherapy. J. Drug Deliv. Sci. Technol..

[86] Shchukin D., Zheludkevich M., Yasakau K., Lamaka S., Ferreira M., Möhwald H. (2006). Layer-by-Layer Assembled Nanocontainers for Self-Healing Corrosion Protection. Adv. Mater..

[87] Zheludkevich M.L., Shchukin D.G., Yasakau K.A., Mohwald H., Ferreira M.G.S. (2007). Anticorrosion Coatings with Self-Healing Effect Based on Nanocontainers Impregnated with Corrosion Inhibitor. Chem. Mater..

[88] Mohammadi M., Salehi A., Branch R.J., Cygan L.J., Besirli C.G., Larson R.G. (2016). Growth Kinetics in Layer-by-Layer Assemblies of Organic Nanoparticles and Polyelectrolytes. ChemPhysChem.

[89] Zheludkevich M.L., Salvado I.M., Ferreira M.G.S. (2005). Sol–gel coatings for corrosion protection of metals. J. Mater. Chem..

[90] Ionov L., Minko S. (2012). Mixed Polymer Brushes with Locking Switching. ACS Appl. Mater. Interfaces.

[91] Luzinov I., Minko S., Tsukruk V.V. (2008). Responsive brush layers: From tailored gradients to reversibly assembled nanoparticles. Soft Matter.

[92] Roiter Y., Minko I., Nykypanchuk D., Tokarev I., Minko S. (2012). Mechanism of nanoparticle actuation by responsive polymer brushes: From reconfigurable composite surfaces to plasmonic effects. Nanoscale.

[93] Lishchynskyi O., Stetsyshyn Y., Raczkowska J., Awsiuk K., Orzechowska B., Abalymov A., Skirtach A.G., Bernasik A., Nastyshyn S., Budkowski A. (2021). Fabrication and Impact of Fouling-Reducing Temperature-Responsive POEGMA Coatings with Embedded CaCO_3_ Nanoparticles on Different Cell Lines. Materials.

[94] Abalymov A., Van der Meeren L., Saveleva M., Prikhozhdenko E., Dewettinck K., Parakhonskiy B., Skirtach A.G. (2020). Cells-Grab-on Particles: A Novel Approach to Control Cell Focal Adhesion on Hybrid Thermally Annealed Hydrogels. ACS Biomater. Sci. Eng..

[95] Fox T.L., Tang S., Horton J.M., Holdaway H.A., Zhao B., Zhu L., Stewart P.L. (2015). In Situ Characterization of Binary Mixed Polymer Brush-Grafted Silica Nanoparticles in Aqueous and Organic Solvents by Cryo-Electron Tomography. Langmuir.

[96] Zhang L., Bei H.P., Piao Y., Wang Y., Yang M., Zhao X. (2018). Polymer-Brush-Grafted Mesoporous Silica Nanoparticles for Triggered Drug Delivery. ChemPhysChem.

[97] Coleman B.R., Moffitt M.G. (2018). Amphiphilic Quantum Dots with Asymmetric, Mixed Polymer Brush Layers: From Single Core-Shell Nanoparticles to Salt-Induced Vesicle Formation. Polymers.

[98] Saveleva M.S., Ivanov A.N., Kurtukova M.O., Atkin V.S., Ivanova A.G., Lyubun G.P., Martyukova A.V., Cherevko E.I., Sargsyan A.K., Fedonnikov A.S. (2018). Hybrid PCL/CaCO_3_ scaffolds with capabilities of carrying biologically active molecules: Synthesis, loading and in vivo applications. Mater. Sci. Eng. C.

[99] Hughes R.A., Menumerov E., Neretina S. (2017). When lithography meets self-assembly: A review of recent advances in the directed assembly of complex metal nanostructures on planar and textured surfaces. Nanotechnology.

[100] Tran K.T.M., Nguyen T.D. (2017). Lithography-based methods to manufacture biomaterials at small scales. J. Sci. Adv. Mater. Devices.

[101] Park W., Rhie J., Kim N.Y., Hong S., Kim D.S. (2016). Sub-10 nm feature chromium photomasks for contact lithography patterning of square metal ring arrays. Sci. Rep..

[102] Lee G., Zarei M., Wei Q.S., Zhu Y., Lee S.G. (2022). Surface Wrinkling for Flexible and Stretchable Sensors. Small.

[103] Yin Y., Liu Z., Song M., Ju S., Wang X., Zhou Z., Mao H., Ding Y., Liu J., Huang W. (2018). Direct photopolymerization and lithography of multilayer conjugated polymer nanofilms for high performance memristors. J. Mater. Chem. C.

[104] Berzina T., Erokhina S., Shchukin D., Sukhorukov G., Erokhin V. (2003). Deposition and Patterning of Polymeric Capsule Layers. Macromolecules.

[105] Kudryavtseva V., Bukatin A., Vyacheslavova E., Gould D., Sukhorukov G.B. (2022). Printed asymmetric microcapsules: Facile loading and multiple stimuli-responsiveness. Biomater. Adv..

[106] Gai M., Frueh J., Kudryavtseva V.L., Mao R., Kiryukhin M.V., Sukhorukov G.B. (2016). Patterned Microstructure Fabrication: Polyelectrolyte Complexes vs Polyelectrolyte Multilayers. Sci. Rep..

[107] Campbell J., Vikulina A.S. (2020). Layer-By-Layer Assemblies of Biopolymers: Build-Up, Mechanical Stability and Molecular Dynamics. Polymers.

[108] Witt M.A., Valenga F., Blell R., Dotto M.E.R., Bechtold I.H., Felix O., Pires A.T.N., Decher G. (2012). Layer-by-Layer Assembled Films Composed of “Charge Matched” and “Length Matched” Polysaccharides: Self-Patterning and Unexpected Effects of the Degree of Polymerization. Biointerphases.

[109] Azinfar A., Neuber S., Vancova M., Sterba J., Stranak V., Helm C.A. (2021). Self-Patterning Polyelectrolyte Multilayer Films: Influence of Deposition Steps and Drying in a Vacuum. Langmuir.

[110] Madaboosi N., Uhlig K., Schmidt S., Jager M.S., Mohwald H., Duschl C., Volodkin D.V. (2012). Microfluidics meets soft layer-by-layer films: Selective cell growth in 3D polymer architectures. Lab Chip.

[111] Yang J., Gao C. (2010). Fabrication of Diverse Microcapsule Arrays of High Density and Good Stability. Macromol. Rapid Commun..

[112] Wang B., Zhao Q., Wang F., Gao C. (2006). Biologically Driven Assembly of Polyelectrolyte Microcapsule Patterns To Fabricate Microreactor Arrays. Angew. Chem. Int. Ed..

[113] Volodkin D., Balabushevitch N., Sukhorukov G., Larionova N. (2003). Inclusion of Proteins into Polyelectrolyte Microparticles by Alternate Adsorption of Polyelectrolytes on Protein Aggregates. Biochemistry.

[114] Volodkin D., Balabushevitch N.G., Sukhorukov G.B., Larionova N.I. (2003). Model System for Controlled Protein Release: PH-Sensitive Polyelectrolyte Microparticles. STP Pharma Sci..

[115] Behra M., Azzouz N., Schmidt S., Volodkin D.V., Mosca S., Chanana M., Seeberger P.H., Hartmann L. (2013). Magnetic Porous Sugar-Functionalized PEG Microgels for Efficient Isolation and Removal of Bacteria from Solution. Biomacromolecules.

[116] Volodkin D. (2014). CaCO_3_ templated micro-beads and -capsules for bioapplications. Adv. Colloid Interface Sci..

[117] Omidi M., Fatehinya A., Farahani M., Akbari Z., Shahmoradi S., Yazdian F., Tahriri M., Moharamzadeh K., Tayebi L., Vashaee D. (2017). Biomaterials for Oral and Dental Tissue Engineering.

